# Graphene Oxide Membranes for High Salinity, Produced Water Separation by Pervaporation

**DOI:** 10.3390/membranes11070475

**Published:** 2021-06-26

**Authors:** Khalfan Almarzooqi, Mursal Ashrafi, Theeran Kanthan, Ali Elkamel, Michael A. Pope

**Affiliations:** Department of Chemical Engineering, University of Waterloo, Waterloo, ON N2L 3G1, Canada; kalmarzooqi@uwaterloo.ca (K.A.); ashrafi.mursal@gmail.com (M.A.); tkanthan@uwaterloo.ca (T.K.); aelkamel@uwaterloo.ca (A.E.)

**Keywords:** 2D materials, membranes, produced water, oil/water separation, pervaporation, graphene oxide, desalination

## Abstract

Oil and gas industries produce a huge amount of wastewater known as produced water which contains diverse contaminants including salts, dissolved organics, dispersed oils, and solids making separation and purification challenging. The chemical and thermal stability of graphene oxide (GO) membranes make them promising for use in membrane pervaporation, which may provide a more economical route to purifying this water for disposal or re-use compared to other membrane-based separation techniques. In this study, we investigate the performance and stability of GO membranes cast onto polyethersulfone (PES) supports in the separation of simulated produced water containing high salinity brackish water (30 g/L NaCl) contaminated with phenol, cresol, naphthenic acid, and an oil-in-water emulsion. The GO/PES membranes achieve water flux as high as 47.8 L m^−2^ h^−1^ for NaCl solutions for membranes operated at 60 °C, while being able to reject 99.9% of the salt and upwards of 56% of the soluble organic components. The flux for membranes tested in pure water, salt, and simulated produced water was found to decrease over 72 h of testing but only to 50–60% of the initial flux in the worst-case scenario. This drop was concurrent with an increase in contact angle and C/O ratio indicating that the GO may become partially reduced during the separation process. Additionally, a closer look at the membrane crosslinker (Zn^2+^) was investigated and found to hydrolyze over time to Zn(OH)_2_ with much of it being washed away during the long-term pervaporation.

## 1. Introduction

The extraction of oil and gas creates a significant volume of oily waste water which is generally known as produced water. The amount of produced water from a given reservoir ranges from less than 1% to more than 80% of the amount of oil extracted depending on the nature of the oil reservoir and its age [1,2]. Furthermore, reports indicate that global oil and gas industries produce about 210 million barrels of water from oil processing per day—an amount that is almost three times the quantity of oil produced worldwide [3,4]. Developing methods capable of efficiently purifying this water for release or reuse are of significant economic and environmental concern [5].

The constituents of produced water rely significantly on the reservoir’s geographic location and geologic formation [6]. Generally, the components of produced water include dispersed oil, dissolved organic compounds, treatment chemicals, bacteria, produced solids, metals, and salts that are often similar to those found in seawater with sodium chloride being dominant. These contaminants pose a high burden to the environment and local ecosystems unless processed and purified before disposal. However, the varied composition makes purification challenging and costly.

Different oil/water separation technologies have been employed in the petroleum industries such as floatation, heating, gravity settling, and centrifugal separators. Additionally, membrane separation has seen growing use over the past decades and is replacing some conventional techniques [7,8]. More common membrane separation technologies include microfiltration (MF), ultrafiltration (UF), nanofiltration (NF), and reverse osmosis (RO). Reverse osmosis is the most dominant technology among other membrane separation technologies in the petroleum industry [8]. However, it is one of the most power consuming separations. It also suffers from fouling problems [9], and swelling-induced failure caused by oil and organic contaminants which are often soluble in polymeric membranes [10,11]. These challenges require that significant pre-treatment steps be implemented prior to the produced water entering the membrane unit.

Nowadays, graphene oxide (GO) is emerging as an exciting membrane material due to its high selectivity and high flux of water transport [12,13]. GO is fundamentally a mono-layer-thick hexagonal honeycomb lattice that bears a large amount of oxygenated functional groups on its basal plane surface and edges which lead to high hydrophilicity and good fouling resistance [14,15,16,17,18]. Furthermore, the flow of selective permeants across a GO membrane occurs through interlayer gaps between the sheets, inter-edge spaces, wrinkles, and surface pores that are created by these functional groups [19]. While fouling can still occur on the hydrophobic regions of the GO basal plane [19], it is often restricted to the surface of the membrane making GO membranes more fouling resistant than polymeric membranes [20].

While GO membranes do not swell significantly in organics or oily dispersions, they do have the tendency to swell in aqueous solutions [21]. This swelling increases the width of the interlayer spaces resulting in poor selectivity and possible delamination and destruction of the membrane [22]. Thus, while GO membranes hold promise in applications such as reverse osmosis (RO), this poor selectivity due to swelling has slowed commercialization and has inspired a significant research effort [23]. For example, to reduce the swelling, engineering the d-spacing between the GO sheets has been accomplished by physical confinement, enabling control over membrane selectivity [24]. In this approach, stacked GO strips were encapsulated using an epoxy to produce physical confined graphene oxide (PCGO). PCGO membranes were then tested for five days and no d-space changing was observed. However, a small reduction in permeation was noted that could be attributed to a partial clogging of the nanochannels. While promising, this method is not capable of producing large area membranes [23]. Various other treatments [22,24,25] for improving the selectivity of GO have been investigated such as the use of crosslinkers with varying chain length, multi-valent cations, etc., but all of these adversely impact the flux and performance of the membrane [25].

One way to bypass this swelling problem is to avoid contacting the GO membrane with water on both sides. One of the oldest membrane technologies used to separate liquids with different volatilities is pervaporation. In pervaporation, a liquid mixture is passed on one side of the membrane and the volatile components are driven through the membrane by a partial pressure difference generated by a vacuum or sweep-gas on the permeate side of the membrane [26,27]. Desalination by pervaporation has recently gained traction as an alternative to RO and membrane distillation processes [28,29] and could provide a promising route to purification of produced water. Liang et al. were the first to demonstrate that unmodified GO membranes supported on polyacrylonitrile (PAN) could be used to effectively desalinate water with >99.8% rejection of salt and high flux (65.1 L m^−2^ h^−1^) when operated at 90 °C, which suggests that swelling of GO is not as challenging in this application due to the presence of a gas phase on one side of the membrane.

**Figure 1:**
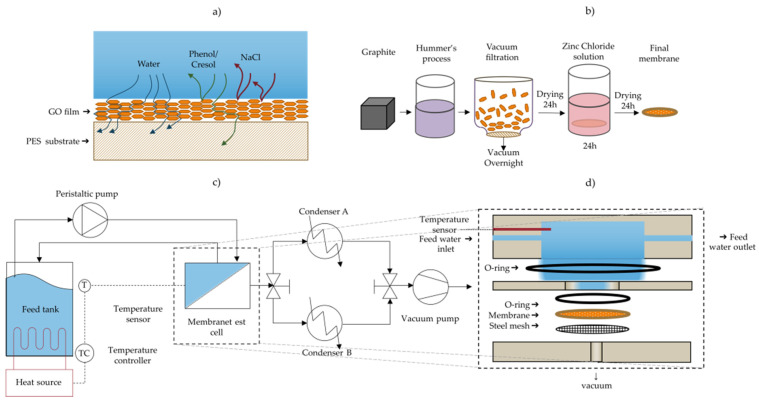
(**a**) Illustration of rejection of chemicals and purification of water via the use of GO membrane pervaporation; (**b**) method of production of GO membranes; (**c**) schematic diagram of the pervaporation separation apparatus; (**d**) schematic of the membrane test cell module showing the flow directions and permeate outlet.

Building upon this work, in this study, we probe the performance of GO membranes for pervaporative separation of a model produced water formulated based on contaminants found in the waste water resulting from the steam-assisted gravity drainage (SAGD) oil extraction process. This study proposes the purification of a produced water model using membranes prepared by vacuum filtered GO sheets on PES films, cross-linked via Zn^2+^ to increase its stability during pervaporation (Figure 1). In addition, we report a long-term study of 72 h of pervaporation. Moreover, a closer look at the membrane’s cross-linker was investigated before and after usage. We find that our membranes significantly outperform those reported by Liang et al. at 30 °C and 60 °C in terms of flux and rejection when tested with water containing 30 g L^−1^ NaCl—despite being thicker. However, the flux is found to drop with increasing amounts of soluble and insoluble organic contaminants. While the membranes do not catastrophically fail after 72 h of processing, the flux and rejection do drop with the membranes continuously able to reject >99% of the salt and initially 55–60% of the dissolved organics. The results suggest that GO membranes may hold significant promise for the purification of produced water.

## 2. Materials and Methods

### 2.1. GO and Membrane Synthesis

GO was synthesized by a modified Hummer’s method [30] as described by Marcano et al. [31]. Briefly, 1.5 g of graphite flakes (obtained from Alfa Aesar, Haverhill, MA, USA, -10 mesh, metal basis, 99.9%) were oxidized at 45 °C overnight via addition to a 9:1 mixture of H_2_SO_4_ (240 mL, Sigma Aldrich, St. Louis, MO, USA) and H_3_PO_4_ (20 mL, Sigma Aldrich) in which 9 g of KMnO_4_ (Sigma Aldrich, St. Louis, MO, USA) was initially dissolved. Afterwards, the solution was transferred to approximately 200 mL of ice water and cooled down. Approximately 5 mL of 30% H_2_O_2_ (Sigma Aldrich, St. Louis, MO, USA) was slowly added while the solution was stirred until a brilliant yellow dispersion was obtained. The resulting GO was then washed of residual acids, etc. by centrifugation (3300 rpm for 30 min, Fisher Scientific, Waltham, MA, USA—acuuSpinTM 3) and redispersion of the pellet twice with 10% HCl (Sigma Aldrich) and then three times with 95% ethanol (reagent alcohol grade, Sigma Aldrich, St. Louis, MO, USA). After the final ethanol centrifuge, the GO was redispersed in ethanol, and the resulting GO solution was kept in ethanol as a slurry, and its concentration was measured. The stock was stored prior to use. Fourier-transform infrared spectroscopy (FTIR- BRUKER) was carried out in order to ensure the successful synthesis of GO from 4000 to 400 cm^−1^ with a resolution of 4 cm^−1^ using a potassium bromide (KBr) disc. Additionally, atomic force microscopy (AFM) was carried out in contact mode (NP-STT10 tips, Nanoscope MultiMode AFM, Veeco) to investigate the exfoliation state of graphene oxide sheets. Dilute dispersions of GO were spun coat from n-butanol onto an ozone treated silicon wafer.

Membranes with three different thicknesses were made by vacuum filtration of varying volumes and concentrations of GO. Using a pipette, 62.7 µL, 89.6 µL, and 134.4 µL were taken from a 6 mg mL^−1^ GO in ethanol stock solution and dispersed in 50 mL of n-butanol (Sigma Aldrich, St. Louis, MO, USA) For each coating, the GO dispersion in n-butanol was tip sonicated at 50% amplitude (Biologics Inc., Manassas, VA, USA, Model: 150V/T). Afterwards, the entire dispersion was vacuum-filtered through a polyethersulfone (PES) filter (0.1 microns, Sterlitech Corporation, Kent, WA, USA) and was left covered (to prevent evaporation) over night. This led to membranes with areal mass loadings of 35 µg cm^−2^, 50 µg cm^−2^, and 75 µg cm^−2^, respectively. The formed GO/PES membranes were dried in a dry air desiccator (Terra Universal, Series-100) at a relative humidity (RH) of 0.5% for at least 24 h.

The GO-coated PES membranes were then immersed in a solution of 100 mL of 0.1 M ZnCl_2_ adjusted to a pH of approximately 5–6 for 24 h using a dilute HCl solution (<1%), in order to crosslink the GO sheets with the divalent Zn^2+^ and enhance the membrane stability in water [22]. Afterwards, the membranes were kept in the dry air desiccator for at least 24 h before testing. The stability of the cross-linked GO was tested by submerging crosslinked and uncrosslinked GO/PES membranes in water under a high speed of mechanical stirring. Figure 1b summarizes the membrane preparation method.

### 2.2. Pervaporation and Membrane Performance

Pervaporation experiments were carried out for five types of solutions: 30,000 ppm (30 g L^−1^) DI water, NaCl solution, single organic constituents (phenol, cresol, or naphthenic acid) in water solution with a concentration as shown in Table 1 with no salt, a laboratory-made produced water model solution, and its composition (as shown in Table 1) [32], and a laboratory made solution of oil foulant mixed with a produced water model solution. Oil foulant solutions were synthesized by dissolving 50 ppm of sodium dodecyl sulfate (SDS, Sigma Aldrich, St. Louis, MO, USA) in DI water that contains 1500 ppm hexadecane (Sigma Aldrich, St. Louis, MO, USA), followed by mixing using a homogenizer (CAT, Model: X-120) for 2 h at 1500 rpm [33]. The resulting emulsion was a white milky dispersion and was stable for at least 24 h (long enough for membrane testing). Afterwards, the produced water model components shown in Table 1 were added to the prepared oil-in-water emulsion.

Pervaporation experiments were carried out using a customized test cell module made from stainless steel (grade 316). Figure 1 illustrates a process flow diagram of the pervaporation separation process used. A rendering of the test cell and flow channel are shown in Figure 1a,d. The feed solution held in a 500 mL flask was heated to either 30 °C or 60 °C using a hotplate. The temperature of the feed solution was controlled by a thermocouple submerged in the aqueous phase inside the test cell and connected to the hotplate, in order to maintain the temperature via the hotplates’ feedback control system. This fluid was circulated to the test cell using a peristaltic pump at a rate of 75 mL min^−1^ (Fisher Scientific), and the retentate was recycled back to the stock feed solution. The effective membrane area exposed in the test cell is 5.07 cm^2^. A vacuum pump (Edwards, Model: E2M-1.5) was used to generate a vacuum of approximately −0.1 MPa (gauge) and the resulting water vapor was collected in one of two glass condensers (Sigma Aldrich, inner cold surface area of 334 cm^2^) cooled to approximately −35 °C with a dry ice/ethanol slurry. Switching between two such condensers with a 3-way valve allowed for continuous operation of the membrane setup and collecting water while measuring the amount of water collected in the other condenser. The mass of the condenser was measured before and after collecting the condensate using a digital balance (OHAUS, Model: NV2101, repeatability = 0.1 g) to determine the permeation flux. The water permeation flux (J) through the membrane was calculated using Equation (1):

(1) $$J=\frac{M}{A\cdot \Delta t},\;(L/m^{2}\;h),$$

where *M* is the amount of water collected, *A* is the effective membrane area, and Δ*t* is the given time interval.

In short-term pervaporation experiments, three independent GO/PES membranes were tested. The feed was circulated through the system at 30 °C for 20 min in order for the test-cell to reach thermal equilibrium. Permeate was then collected for 2 h in approximately 20-min intervals. The temperature was then raised to 60 °C and left for 1 h to reach thermal equilibrium. Afterwards, permeate was collected for 2 h in approximately 20 min intervals. The average flux and rejection for each temperature or test solution was calculated and the error was approximated as ± the standard deviation.

In longer term studies, the various feed compositions were examined for 72 h of permeation using the 50 µg cm^−2^ GO/PES membrane. After the test cell reached thermal equilibrium (20 min), sample collection was carried out every 1 h.

The Nelson method was used to indicate outliers in permeate flux which were defined as data points more than three standard deviations away from the mean. Additionally, one-way ANOVA tests were performed to determine whether the differences in fluxes and rejections are statistically significant by figuring out its *p*-value.

Water samples collected in the condensers were analyzed for salt concentration and produced water model solutes, by measuring the conductivity and by using ultraviolet-visible spectroscopy (UV-Vis, Thermo Scientific Evolution 300), respectively, before and after separation runs. Permeate conductivity measurements were carried out using an OAKTON (PC700, Vernon Hills, IL, USA) conductivity meter. For UV/vis, a set of known solution concentrations were prepared to establish a concentration calibration curve with their respective intensities at wavelength of 269 nm for phenol and 271 cresol, in order to measure their respective concentrations in the collected samples. For naphthenic acid, produced water model (PWM) and produced water model with foulants solution, the absorption intensities of the feed, and collected samples were measured at 265 nm for naphthenic acid, and 270 nm for PWM and PWM with foulants’ solutions. The resulting UV/vis curves were normalized in order to investigate any changes in the absorbed peaks and were compared to the starting feed intensity peaks. The solute rejection was calculated using Equation (2):

(2) $$R=\left(\;1-\frac{C_{p}}{C_{f}}\;\right)\times 100\%,\;(\%),$$

where *C_f_* and *C_p_* are the collected and the initial solute concentration, respectively. 

The change in hydrophilicity of the membrane due to chemical changes or the adsorption of simulated produced water model foulants was carried out using the sessile drop method and a smart-phone camera carefully positioned normal to the substrate plane [34]. A drop of DI water was added vertically using a pipette to the surface of the membrane, and a photo was taken directly. The droplet was further analyzed using ImageJ software. The contact angle of three droplets placed on different areas of the same membrane were used to calculate the average contact angle and the standard deviation was used to approximate the error. All membranes tested for contact angle were left to dry for at least 24 h after the pervaporation test was done. Membranes of 50 µg cm^−2^ GO specific deposition were tested before and after the long-term pervaporation experiment for all three of the tested solutions. Powder X-ray diffraction (XRD, Rigaku Miniflex II) was acquired using a range of 2θ between 5° to 65° using Cu kα radiation (λ = 1.54059 Å). A thermal emission scanning electron microscope (SEM) imaging was done by a TESCAN Vega instrument with voltage acceleration of 10 kV using a secondary electron detector. Samples were sputtered with gold for 139 s to render the surface of the membranes conductive for imaging. The thickness of the GO layer was estimated from a cross sectional image using several measurements by ImageJ software.

Elemental composition was carried out by XPS for unused (control) and used membranes for long periods of pervaporation (72 h). The XPS analysis was carried out using (Thermal Scientific KAlpha XPS spectrometer) operated in ultra-high vacuum (2 × 10^−10^ mbar) with acquisition time of 0.2 s. Survey spectra and high resolution scans for C 1s, O 1s, and Zn 2p spectra were obtained. The distribution of the C and O groups at the membrane surface were identified by deconvolution of the spectra peaks using CASAxps software, and all the peaks were shifted based on the C-C peak (284.5 eV) and using a Shirley background removal.

## 3. Results and Discussion

### 3.1. GO Membrane Characterization

**Figure 2:**
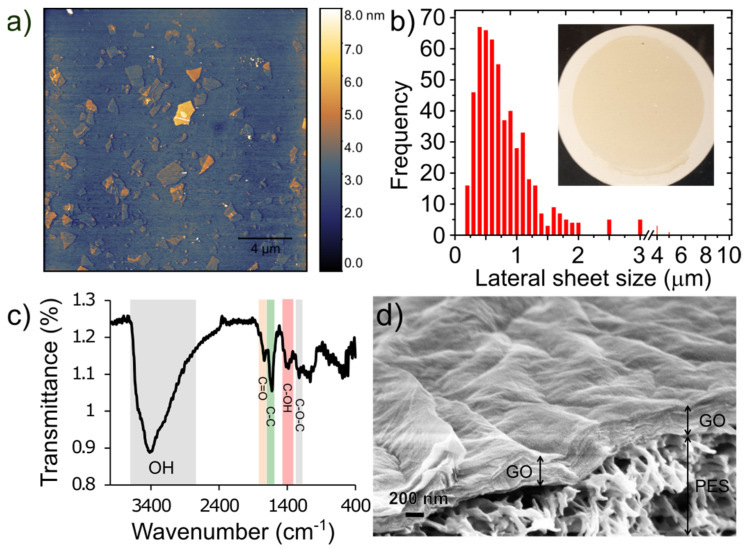
(**a**) AFM images of graphene oxide flakes spun coat onto Si wafer, (**b**) histogram of lateral sheet size distribution. The inset shows a photo of a 50 µg cm^−2^ GO/PES membrane prepared by vacuum filtration. (**c**) FTIR spectrum for GO sheets; and (**d**) cross-sectional SEM image showing surface topology and thickness of a 50 µg cm^−2^ GO membrane and the underlying PES support.

Figure 2a shows AFM images of the exfoliated GO sheets. The results show that most of the GO flakes are atomically flat, unwrinkled, and comprised of single layers with thickness of approximately 1 nm. A histogram of the lateral sheet size distribution is shown in Figure 2b illustrating that the majority are ~0.5 µm in size with a tail in the distribution showing some sheets as large as 1–10 µm. Figure 2c shows the FTIR spectrum of the synthesized graphene oxide. Several characteristic peaks for GO are observed including a broad peak between 3600 cm^−1^–2400 cm^−1^, which is attributed to stretching vibrations of O–H groups, a sharp peak at 1740 cm^−1^ attributed to the C=O stretching vibration, a sharp peak at 1623 cm^−1^ due to unoxidized sp^3^ C-C bond, a peak at 1415 cm^−1^ due to C–OH deformation, and 1227 cm^−1^ due to C–O–C vibrations [31,35]. An SEM image of the top surface and cross section of a GO/PES membrane is shown in Figure 2d. The GO layer thickness is estimated to be 466 ± 52 nm. This is thicker than what would be expected based on the loading and assumed full density of GO (~1.8 g cm^−3^) which for 50 µg cm^−2^ would lead to a fully dense thickness of 278 nm. It is known that vacuum filtration yields somewhat disordered films compared to a technique like pressure-assisted vacuum filtration. A more ordered film is likely to form early in the vacuum filtration process when the flow is high while evaporation of the solvent at the slower stage of filtration may cause the upper portion to become more disordered. For example, Tsou et al. [36] demonstrated a factor of 384 nm/231 nm = 1.66 difference between vacuum filtered GO films and nearly fully dense films prepared by pressure-assisted vacuum filtration, which is nearly the same ratio we observe above (1.67).

**Figure 3:**
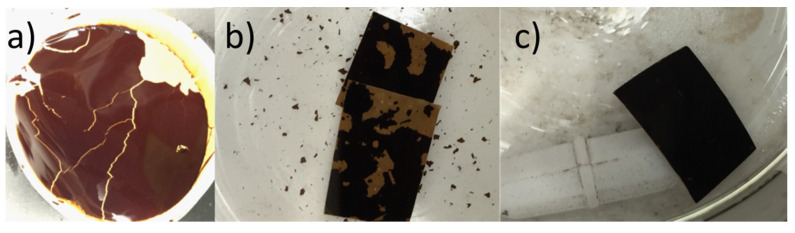
Membrane stability enhancement by Zn^2+^ crosslinking: (**a**) vacuum filtered GO membrane immersed in water for 30 min; (**b**,**c**) squares of membrane exposed to high speed mechanical stirring with no zinc treatment: squares in (**b**) have no Zn^2+^ treatment while the membrane in (**c**) was soaked in the Zn^2+^ solution for 24 h.

**Figure 4:**
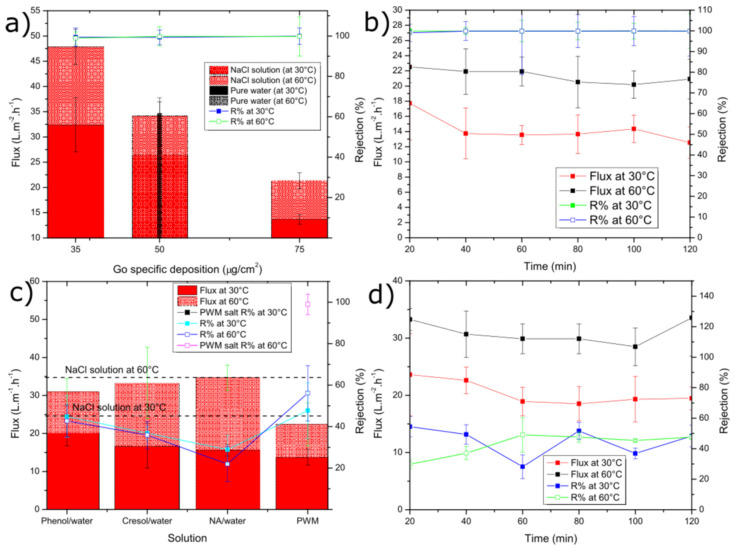
Average water flux and solute rejection for: (**a**) NaCl solution and pure water at different GO loading; (**b**) NaCl solution tested by 75 µg cm^−2^ GO loading; (**c**) single organic and PWM solutions; (**d**) single organic (phenol solution). For (**c**,**d**), a 50 µg cm^−2^ GO loading was used. The error bars were estimated as ± one standard deviation of three independent measurements.

To improve the stability of the various GO membranes, the GO was cross-linked with Zn^2+^ by soaking the membranes in a solution of ZnCl_2_. Figure 3 compares the stability of vacuum-filtered GO/PES membranes before and after cross-linking and after exposure to high-speed mechanical stirring of water. Without cross-linking, the GO began to flake off the substrate while the cross-linked GO remained intact. The good mechanical strength of cross-linked GO sheets was first reported by Yeh et al. [22], where they discovered that Al^3+^ originating from AAO filter discs was the cause of the mechanical property enhancement of vacuum cast films using this popular but non-scalable membrane support. In addition, the GO functional groups like hydroxyl and carboxyl groups can be used with different metallic cross-linking agents to create a coordination bond, and thus it can be used to increase the membrane’s stability and tune the d-spacing between GO sheets [25]. These metallic cross-linking agents include divalent cations like: Ni^2+^, Mg^2+^, and Ca^2+^, or trivalent cations like: La^3+^, Fe^3+^, and Al^3+^ [25,37]. Moreover, Yu et al. [31] listed the ions’ stability capabilities as follows: Al^3+^ > Ca^2+^ > Mg^2+^ > Na^+^. This has since become a common method to enhance GO membranes for nanofiltration applications but has not been studied using pervaporation. Membranes that were not enhanced by Zn^2+^ crosslinking were found to be unstable and caused frequent leaks in our pervaporation cell. As shown in Figure 4, the rejection for all 30 g/L NaCl samples was higher than 99.31%.

### 3.2. Short-Term Membrane Performance

Figure 4a shows the average water permeation flux and salt rejection through the GO membrane for tests carried out at both 30 °C and 60 °C using membranes of different GO loadings (i.e., thickness). The flux for pure water through the 50 µg cm^−2^ membrane at 30 °C was 31.6 ± 1.3 L m^−2^ h^−1^, which was slightly higher than the solution containing 30 g L^−1^ NaCl (26.5 ± 2.7 L m^−2^ h^−1^). Our GO membranes outperform those reported by Liang et al. [38], who measured a flux at 30 °C only as high as 14.3 L m^−2^ h^−1^ for 35 g L^−1^ NaCl with an even thinner membrane (1.8 µg cm^−2^). This improvement might be attributed to differences in the GO synthesis method used or the fact that we use a Zn^2+^ cross-linker, which could increase the d-spacing. They also reported a decrease in flux with increasing salt concentration in the range of 2000–100,000 ppm but did not test under pure water. The drop in flux is likely due to a decrease in the concentration and activity of free water vs. water bound to the salt ions as a hydration shell. The flux was found to increase with temperature in all cases with a more significant increase for the NaCl cases vs. pure water. This also confirms the results of Liang et al. who illustrated a positive activation energy for water transport and the Arrhenius-like temperature dependence of permeance for their GO membranes. The flux was also found to be almost linearly dependent on decreasing membrane thickness. While thinner membranes could be fabricated, we found that the 35 µg cm^−2^ had a low failure rate compared to thinner ones. As shown in Figure 4b), the permeate flux and rejection remained relatively constant over the 2 h pervaporation run.

Next, we introduce several of the model contaminants used in our PWM to study their impact on flux and the GO membrane’s ability to reject these contaminants. As shown in Figure 4c), at both 30 °C and 60 °C, the flux through the membrane drops slightly compared to the NaCl only case. However, it is not found to change significantly (as determined by ANOVA with *p* > 0.05) as a function of either the single or mixed organic contaminants. The decreased flux can also be explained by the additional solutes added, which would act to decrease the activity on the feed side as discussed previously. However, since the single organic solutions contain no salt and these mixtures have less dissolved solute, it is more likely that the contaminants may adsorb to the GO within the membrane and inhibit water transport. Figure 4c also shows that the rejection of each contaminant is similar within error (as determined by ANOVA with *p* > 0.05) and is ~40% irrespective of temperature. The reason for this could be the similar molecular structure. Phenol and cresol have a similar aromatic structure, except cresol has an extra methyl (-CH_3_) group, and NA has a longer chain of repeated aromatic groups, but it was used at lower concentration. An example of a single organic component (phenol) flux vs. rejection is given as shown in Figure 4d. Both values were fairly constant over the 2 h run.

**Figure 5:**
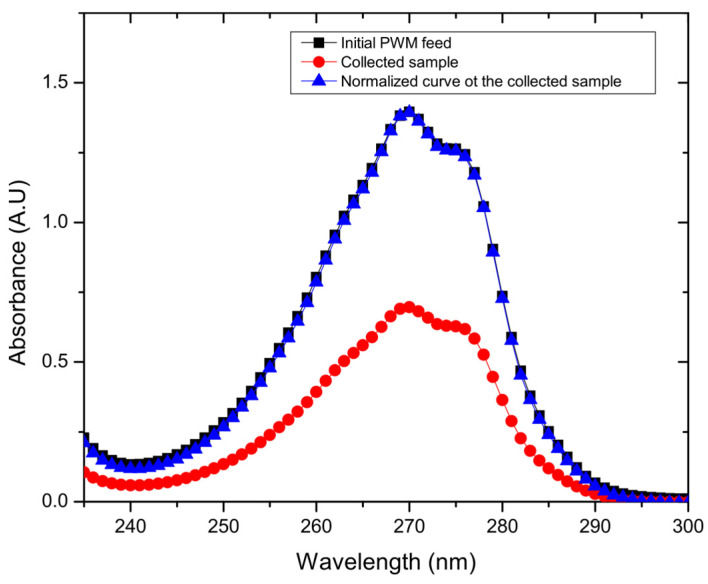
Example UV/vis spectra for one of the collected samples, and its normalized curve matching the initial PWM curve.

As also shown in Figure 4c, the water permeation flux of combined PWM through the GO membrane shows no significant difference in flux when compared to the single organic components at 30 °C as ANOVA testing reveals a value of *p* > 0.05. The flux is 13.8 ± 2.1 L m^−2^ h^−1^, with a rejection of 47.7% ± 13.5%. However, there was a significant difference with the higher temperature tests at 60 °C, which exhibited slightly lower flux of 22.4 ± 5.7 L m^−2^ h^−1^ and rejection of 56.1% ± 13.3% as ANOVA testing shows a *p* < 0.05. As shown in Figure 5, when all PWM contaminants were added together, the permeate collected had a nearly identical UV/vis plot but scaled down in intensity. Normalizing the plot reproduced the original mixture which suggests that all components were collected in the same proportion as in the feed but at a reduced concentration. Thus, the rejection in this case could be easily calculated from the overall UV/vis results without having to deconvolute contributions from the individual components. Thus, all contaminants whether present together or individually are rejected to a similar extent (~40–55%) by the GO membrane. These results demonstrate that, while being able to effectively desalinate the produced water, the membrane may also be used to purify produced water from residual soluble organic components with moderate selectivity.

Moreover, the water permeation flux for PWM is generally lower than that of NaCl solution and single organic component solution, and presented results with a higher standard deviation throughout the experiment. For example, the relative error for the NaCl only case is 10% and 8% for 30 °C and 60 °C, respectively, while, for the PWM, was 15% and 25% for 30 °C and 60 °C. The reason behind that is the continuous adsorption and desorption of the PWM solutes on the membrane surface which may act to block some of the nano-scale channels within the GO membrane’s pores.

### 3.3. Long-Term Pervaporation Study

**Figure 6:**
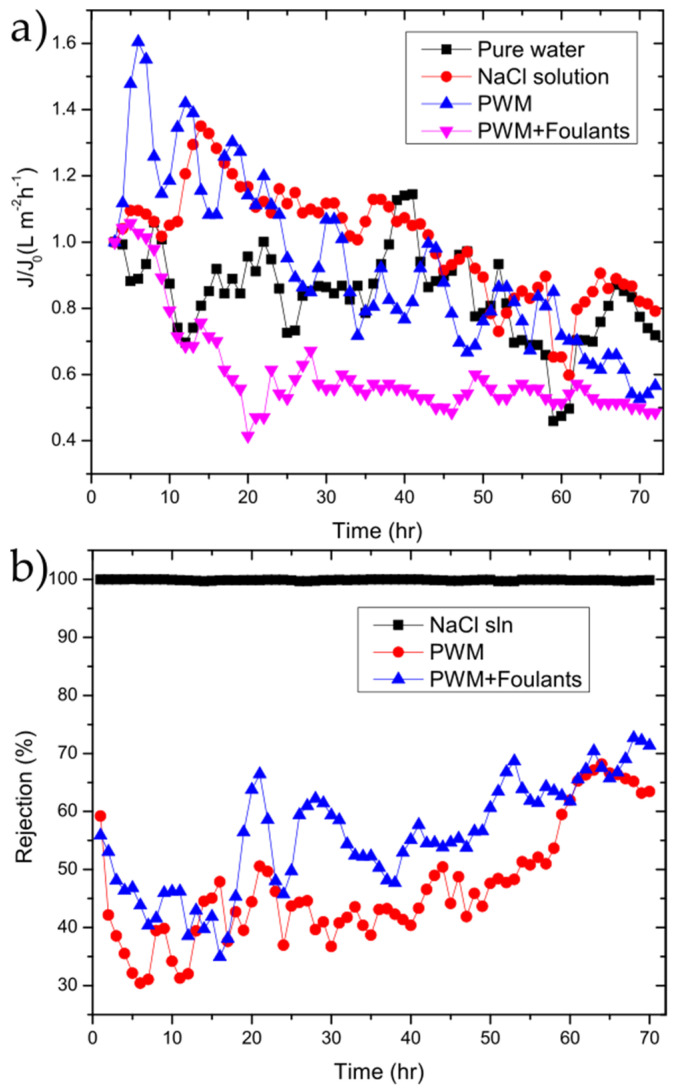
(**a**) flux/flux_0_ (J/J_0_); (**b**) and rejection vs. time for 50 µg cm^−2^ GO membrane for the tested solutions.

The GO membranes were also tested to investigate their performance over longer periods of time using the NaCl solution, PWM, and PWM with foulants along with the control solution of water as shown in Figure 6.

The water flux followed the same general trend as shown in Figure 4b. To better highlight the changes as a function of time, the flux normalized to the initial flux is plotted as in Figure 6b. In all cases, there is a drop in flux over the 72 h period with the PWM and PWM with foulants exhibiting a larger drop of 50–60% of the initial while the pure water and NaCl solutions retain 70–80% of their initial flux. This is likely due to both the continuous build-up of foulants within the GO selective layer and concentration polarization near the membrane surface, which is more severe in the PWM cases. However, it could be due in part to the partial deoxygenation of the GO in water as will be discussed later. The rejection for solutions of the PWM and PWM with foulants slightly increased with time. This could be attributed to pore plugging by the foulants caused by the direct adsorption of contaminants onto the membrane surface.

**Figure 7:**
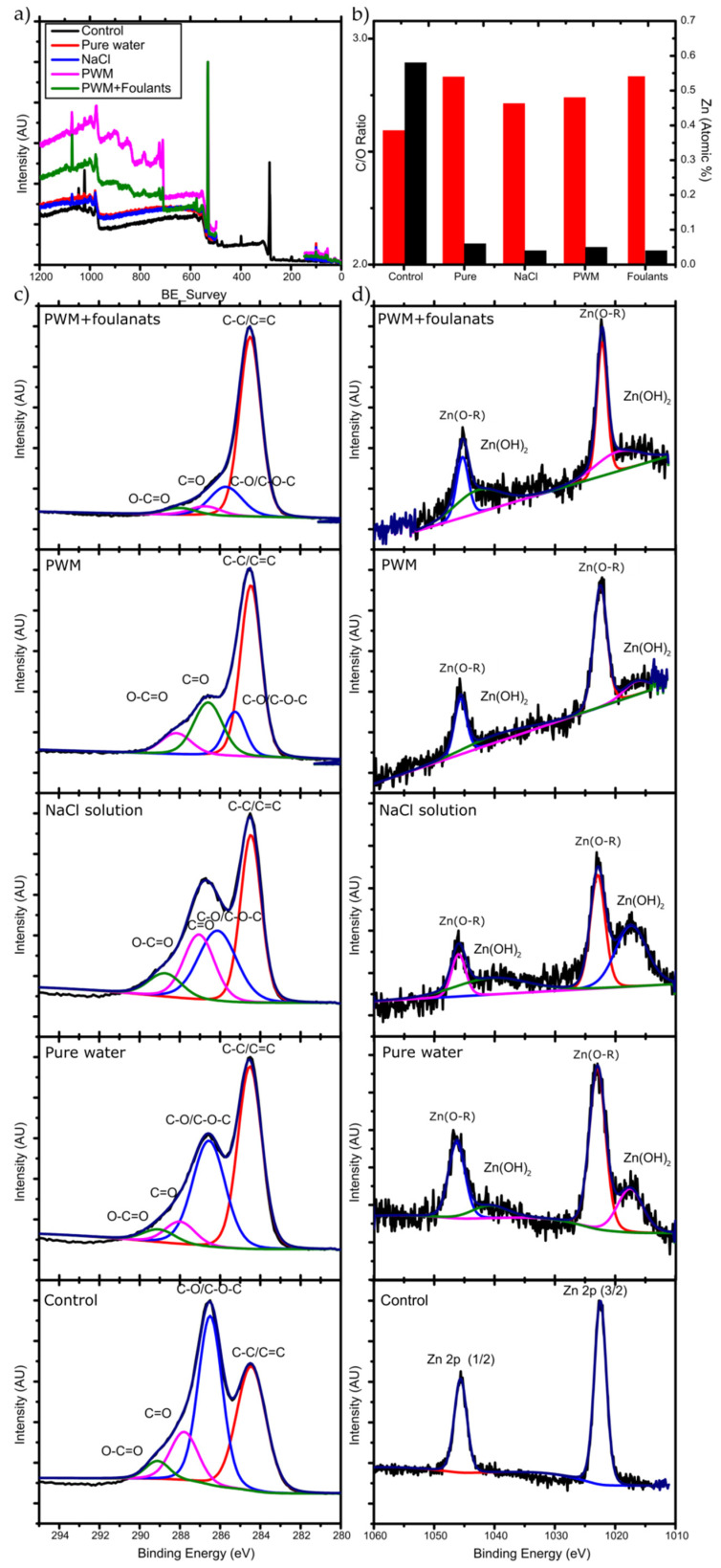
(**a**) XPS survey data; (**b**) C/O ratio (red bars) and Zn atomic % (black bars), high resolution XPS for (**c**) C 1s and (**d**) Zn 2p for the unused and used membranes for different solutions.

The membranes used in the longer-term PV tests were removed from the test cell and further characterized to determine any chemical and structural changes resulting in the decreased flux observed. To further understand the changes in flux over the 72 h pervaporation, we carried out XPS and measured the contact angle of water on the used membranes. Survey spectra and high resolution C 1s, O 1s, and Zn 2p spectra were obtained. Figure 7 demonstrates the XPS scan spectrums obtained along with the peaks’ deconvolution analysis and the C/O ratio analysis. The binding energies for the carbon, oxygen, and zinc were identified, and their atomic percentages were calculated.

The deconvolution of C 1s revealed four individual peaks of C-C, C-O, C=O, and O-C=O and their relative percentages are shown in Table 2. This reveals a higher content of C-C bonding and lower C-O content for all of the used membranes compared to the unused control.

The corresponding change in C/O ratio is illustrated in Figure 7b. The membrane that was used for pure water pervaporation has the highest C/O ratio after the 72 h run. Since there is no organic materials present in the feed, the increase must be due to the deoxygenation of GO upon exposure to water. It is well known that the chemical structure of GO can evolve upon exposure to water, which has been described by the Tour group’s dynamic structural model (DSM) [39]. In this model, the structure of GO is not static and will continuously change when it reacts with water, introducing new oxygen containing functional groups and leaving the GO structure by different mechanisms. New protons (H_3_O^+^) are created from the acidic groups on GO, making the GO more negatively charged, and stabilized by the resonance of the oxygen containing groups and by the formed electric double layer near the surface.

**Figure 8:**
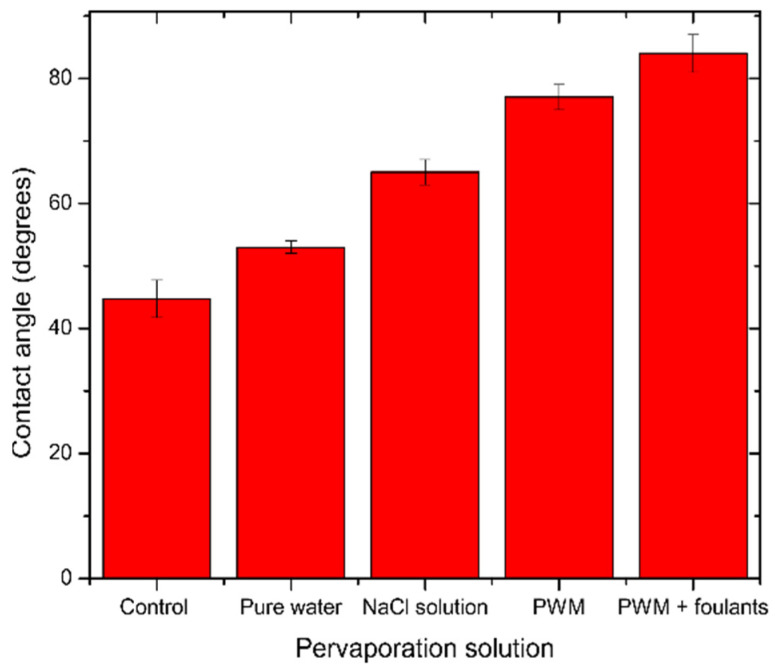
Average contact angle for unused and used GO/PES membranes tested for different solutions. The error bars were estimated as ± one standard deviation of three independent measurements.

In the case of NaCl solution pervaporation, Na+ ions will be loosely associated near the GO surface by coulombic forces and will compete with H_3_O+ ions. However, Dimiev et al. [39] found that the presence of NaCl will change the orientation of the water molecules to be away from the GO surface, and therefore less deoxygenation happens to the GO, which is also observed in Figure 7b by a less severe change in C/O ratio as with pure water. In the case of PWM and PWM with foulants, these solutions have the same concentration of NaCl, and, therefore, their higher C/O ratio when compared to NaCl solution is likely due to the deposition of the organic materials on the surface which increased the C-C content and blocked the pores from the water, causing further deoxygenation. Furthermore, the PWM with foulants shows a relatively higher content of O-C=O as shown in Table 2. This may be attributed to naphthenic acid that remains adsorbed to the GO. These findings are all in agreement with the contact angle measurements as shown in Figure 8.

Moreover, Figure 7b shows the deconvolution of the Zn 2p spectra. There are two strong peaks around 1021.3 and 1044.5 eV that are attributed to the binding energies of Zn 2p_3/2_ and Zn 2p_1/2_ of the Zn(O–R) interaction caused by the crosslinking between adjacent GO. After 72 h of pervaporation, these initial peaks each split into two broad peaks. The lower binding energy deconvoluted peaks represent the Zn^2+^ of the hydroxide (lower intensity), and the higher binding energy of the deconvoluted peaks represents the Zn(O–R) interaction (higher intensity) [40]. The Zn(OH)_2_ was formed due to the hydrolysis of the Zn/GO organometallic complex during the pervaporation as illustrated in Equation (3) (reproduced from [41]).
(3)
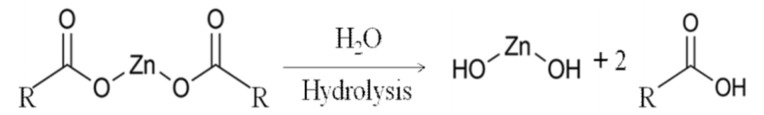
.

The amount of Zn present before and after membrane testing is shown in Table 3. A reduction of the Zn-crosslinker is observed after 72 h of pervaporation. XPS Zn 2p spectra suggests that more degradation happened to the Zn(O-R) cross-linker for the membranes used for pure water and NaCl solution due to a higher peak intensity of Zn(OH)_2_ compared to Zn(O-R) peak intensity. This is likely due to the higher flux of these membranes which hydrolyzes more of the Zn to Zn(OH)_2_, which may also act to reduce the membrane stability over longer time exposure to water.

In addition, different elements were observed from the survey spectra at the surface of the membrane; these elements include Na, Cl, and S. Table 3 shows the atomic percentage (%) on each tested membrane. In the pure water tested membrane, no Na or Cl elements were observed, since there were likely to be washed away from the surface after a long period of pervaporation. The high S content in the PWM with foulants membrane is likely coming from the SDS that was added to the solution as a foulant.

TGA was attempted to investigate the nature of foulants on the membrane, but the loading of the GO on the PES is too thin and its mass nor the mass of other adsorbed organics and foulants could be detected. The GO forms only around 0.7% of the overall weight of the membrane.

While produced water separation has not been studied by pervaporation with pure GO-based selective layers, it has been studied with several mixed-matrix membranes. In a work done by Alammar et al. [42], a high permeation flux (91.3 L m^−2^ h^−1^ bar^−1^) and rejection (99.96%) were achieved using a thick GO/rGO and PBI matrix membrane for their simulated produced water model. Moreover, randomly arranged GO sheets in the GO membrane by vacuum filtration, and cross-linked by polyethyleneimine (PEI) was used to for oil water separation by gravity filtration [43]. In their work, they achieved as high a permeation flux as 688 L m^−2^ h^−1^ of a simulated oil-in-water emulsion of water in hexane (99:1). Additionally, graphene/polyvinylidene fluoride (G/PVDF) used for produced water–air gap membrane distillation (AGMD) [44], and it showed a high permeation flux of (20.5 L m^2^ h^−1^) and salt rejection (99.99%) for optimal graphene loading of 0.5 wt%. Furthermore, the long-term study of 10 days showed that incorporating graphene in the PVDF membrane will enhance the robustness of the membrane and wetting resistance when compared to commercial PVDF. We can compare our results and stability to some recently published literature on desalination and produced water separation using other technologies. Recently, Liu et al. [45] used MXene-based membranes for pervaporation desalination and demonstrated fluxes as high as 120 L m^−2^h^−1^ at 65 °C for their thinnest membranes. While this is slightly higher than what we report for the salt solution case, their long-term testing, also carried out at 30 °C, resulted in a permeance of < 3.5 L m^−2^ h^−1^. Our membranes, even with the additional PWM contaminants, achieved a very similar level of performance. In even more recent work, impressive permeance and selectivity have been achieved with cross-linked polymer systems such as cross-linked polyvinylalcohol/PAN [46]. However, these polymer systems have not been tested with produced water and may foul quite rapidly.

## 4. Conclusions

In this study, graphene oxide (GO) membranes were prepared by vacuum filtration on polyethersulfone (PES) and cross-linked using divalent zinc cations to enhance their stability. The prepared membranes were tested for desalination and produced water separation using a simulated SAGD process model. Short- and long-term studies were carried out on three different GO loading membranes to investigate the permeation flux and solute rejection. The initial fluxes were significantly higher than those reported previous for GO membrane-based desalination, but the flux gradually reduced as increasing solutes and foulants were added to the water. While the rejection for salt was found to be ~99% in all cases, the rejection of soluble organic components (phenol, cresol, napthanic acid) was in the range of 40–50%. The flux and rejection slightly reduced over 72 h of membrane operation. The flux for the pure water, salt, and simulated process water gradually decreased to a steady-state of 70%, 80%, and 50%, respectively. In addition, this study highlighted the degradation of the Zn^2+^ crosslinker used to hold the GO sheets together. After 72 h of pervaporation, the examined membranes showed a lower amount obtained for Zn^2+^ and a hydrolysis transformation to Zn(OH)_2_. This is an important aspect to focus on in determining the membranes stability over a longer period of usage. While this flux is in the same range as what has been reported for the best performing MXene membranes used for pervaporation-base desalination, future work will focus on stabilizing the high initial flux we observe in our GO membranes while reducing the thickness without compromising mechanical stability. We hope that these results shed light on the promise of using pervaporation and graphene oxide membranes as a tool for more economical produced water separations.

## Figures and Tables

**Table 1:** Composition of produced water model solution, based on steam assisted gravity drainage (SAGD) process [32].

Component	Concentration (ppm)
Phenol	45
Cresol	45
Naphthenic acid	10
Sodium chloride	30,000

**Table 2:** Relative contribution (%) of the deconvoluted C 1s peaks for unused and used membranes corresponding to different bonding environments.

Peaks	Control	Pure Water	NaCl Solution	PWM	PWM + Foulants
C-C	38.67	49.2	39.08	53.73	72.94
C-O	42.72	38.66	30.08	13.8	17.58
C=O	14.1	7.54	22.27	9.6	5.31
O-C=O	4.51	4.6	8.57	22.87	4.17

**Table 3:** Atomic% of Na, Cl, S, and Zn elements observed on the membrane surface.

Element	Control	Pure Water	NaCl Solution	PWM	PWM + Foulants
S	0.43	0.25	0.22	0	3.23
Na	1.69	0	1.01	2.3	1.79
Cl	0	0	0.44	0.86	1.22
Zn	0.56	0.06	0.04	0.05	0.05
